# South West Orthopaedic Club

**Published:** 1991-12

**Authors:** 


### South West Orthopaedic Club

### Meeting at Hereford, October 1991

**The morning session consisted of clinical cases an X-ray Quiz, Poster and video demonstrations and a Trade exhibition. In the abstracts that follow the author who presented the paper is marked with an *. The last two abstracts were poster demonstrations and the remainder were clinical papers.**
**The X-ray quiz was won by Mr. D. Halpin of Torbay and the SWOC Prize certificate was won by Miss D. Eastwood of Bristol. The Pridie Memorial lecture entitled "South West Orthopaedics — 1948-91" was given by Mr. A. H. C. Ratliff of Bristol.**

### ANAESTHESIA FOR HIP AND KNEE REPLACEMENT

***J. Balance**
Anaesthetist, Hereford

The Anaesthetist can help in the prevention of potentially fatal complications from major joint replacement. The "standard" anaesthetic for joint replacement has been a general anaesthetic with or without paralysis and ventilation, usually involving intermittent positive pressure ventilation.

Deep venous thrombosis and pulmonary embolus are very common after orthopaedic surgery, particularly operations on the lower extremities, and may be as common as 80% after total hip replacement. They are the main post-operative cause of morbidity and mortality. Most predisposing factors can be excluded and care in surgery ensures that the risk is minimalised. Prophylaxis is used and there are many regimes. Claims are made for the efficacy of each regime.

Deep venous thrombosis can be reduced from around 70% to less than 15% by the use of an epidural for total hip replacement and the incidence of pulmonary embolus can be brought down to around 10%. This is because of increased circulation in the lower extremities, less activation of the clotting mechanism, and more efficient fibrinolysis. Regional blockade also confers the advantages of easier surgery, stress-free surgery, greater patient comfort post-operatively, better post-operative pulmonary function, less mental dysfunction and less blood loss. It is also likely that an epidural helps the cement/bone interface to be better, leading to longer prosthesis life.

General anaesthetic with spontaneous ventilation will cause some lowering of the venous pressure and thus of the venous channels in the bone of the lower limb. General anaesthetic with paralysis and intermittent positive pressure ventilation **raises** the venous pressure and, it is postulated, makes the cement/bone interface less effective.

An epidural, particularly when prolonged into the post-operative period, causes a marked **reduction** in the venous pressure and allows better joint cementing.

Cement/bone interface apart, workers from Uppsala in Sweden have stated that "epidural analgesia, prolonged into the post-operative period, in addition to other thrombo-prophylactic measures, should be of value in patients undergoing operations associated with a high risk of thromb-embolic complications".

### SHOULD ANKLE FRACTURES IN THE ELDERLY BE FIXED? (Paper)

***W. J. Leach, M. J. F. Fordyce**
Truro

To assess the results of ankle fracture fixation in the elderly, we have carried out a review of seventy-six patients aged over fifty, all of whom had ankle fracture fixation carried out in the two years 1989-1990.

Case notes and pre- and post-operative X-rays were studied, in particular with respect to the early complications of operative treatment.

Nine of the seventy-six patients (11.8%) developed one or more complications. Our series had a 1.8% incidence of infection and a 5.2% incidence of delayed healing and wound necrosis. Malunion occurred in 7.9% of patients, and was due in all cases to imperfect per-operative reduction or inadequate fixation. In no case did fixation fail because of poor bone quality. There was no significant difference between the complication rates in males and females.

We conclude that internal fixation of ankle fractures in the elderly patient carries acceptable risks, so long as careful attention is paid to surgical technique, and fixation is in accordance with AO principles.

### THE LONG TERM PROGNOSIS OF DISPLACED COLLES' FRACTURE: A TEN YEAR PROSPECTIVE REVIEW (Paper)

***J. Field, D. Warwick, G. C. Bannister, A. G. F. Gibson**
Southmead Hospital, Bristol

In 1981 100 consecutive patients with displaced Colles' fracture were treated by closed reduction and cast immobilisation for five weeks. Patients were assessed clinically and radiologically after five weeks, 12 weeks and 10 years. 55 patients attended for follow up after ten years, 35 patients had died.

85% of patients had a clinically satisfactory result after ten years. Significantly more patients had an excellent wrist after ten years. At long term follow up 56% were pain free, 40% had a reduced grip strength; 64% showed objective cosmetic deformity and features of algodystrophy were present in 27%. Osteoarthrosis was present in 45% of injured wrists after ten years, but the prevalence and severity was comparable in the uninjured wrist. Osteoarthrosis occurred more frequently in intra-articular fractures.

**Conclusion**
85% of Colles' fractures are clinically satisfactory after ten years. Clinical results improve little after three months. Displaced intra-articular fractures, wrist deformity and algodystrophy are associated with an unsatisfactory outcome.

### EFFECT OF HETEROTOPIC OSSIFICATION FOLLOWING CLOSED FEMORAL NAILING ON WEAKNESS OF HIP ABDUCTION (Paper)

***A. Biyani, D. A. Jones, C. L. Daniel, M. Bishay**
Morriston Hospital, Swansea SA6 6NL

**Absract**
We analysed the relationship of peritrochanteric heterotopic ossification with the strength of hip abduction following closed intramedullary nailing for femoral shaft fractures. 25 patients with an average age of 21.8 years (range 16-74 years) were reviewed 9 months to 4 years (mean 2 years) post operatively, and weakness of hip abduction as compared to normal hip was assessed using a force transducer (BBC X7 Servigon). Heterotopic ossification was noted in 21 (89%) patients and was graded according to Brumback (1990) criteria.

Three groups were identified:

a) 8 patients did not have any abductor weakness
(3 Grade 0, 3 Grade I, 2 Grade II heterotopic ossification)
b) 8 patients had slight weakness (8-20%) of hip abduction, but were clinically normal
(1 Grade I, 2 Grade II, 5 Grade III heterotopic ossification)

c) 9 patients had significant (32-80%, mean 48.6%) weakness of hip abduction and were variably symptomatic (1 Grade 0, 4 Grade I, 3 Grade II and 1 Grade III heterotopic ossification).

Grade III heterotopic ossification is usually associated with varying degree of weakness of hip abduction. Patients with significant weakness usually have associated contributory factors i.e. severe ipsilateral fractures and a long intramedullary nail.

### COMPOUND LOWER LIMB FRACTURES IN CHILDHOOD: A COMPLEX PROBLEM

***Patricia Allen and Deborah Eastwood**
Frenchay Hospital, Bristol

Compound fractures in childhood occur rarely but they pose complex problems for doctors involved in their management. We have reviewed 8 children who had sustained Grade 2/3 compound fractures of the lower limb, distal to the knee. The average age of the patients was 10 years (range 7-15) and all sustained their injuries in road traffic accidents. The injuries consisted of 6 fractures of the tibia/fibula and 2 ankle/foot injuries. Six cases had an external fixator applied. The soft tissue procedures performed were: 5 fascio-cutaneous flaps, 1 vascularised free fibular graft and 2 free muscle flaps. In 5 cases soft tissue coverage was achieved rapidly, within 1 week, and in these patients there were less problems with swelling, infection and healing of the flaps. In 5 cases the fracture has united uneventfully, in 1 there is a delayed union with a painful fracture site at 5 months and in 2 cases, at 3 month follow up, union appears to be progressing. None of the injuries have so far been associated with any growth disturbances. We believe that children with these injuries should be treated aggressively with early external fixation of their fractures and prompt soft tissue coverage.

### TRANSIENT SYNOVITIS OF THE HIP: AN IRRITATING EXPERIENCE

***Deborah M. Eastwood**
Bristol Royal Infirmary

In 1986, a retrospective review of children admitted to a Hospital with a painful hip was conducted. During 30 months there were 181 admissions, 165 with transient synovitis (TS). Although 25% had a raised white count, 39% a raised ESR and 21% were pyrexial, no hip initially diagnosed as TS developed septic arthritis. 100 hips underwent an ultrasound scan and joint effusion was demonstrated in 54%. Management consisted of traction for those with an effusion and symptomatic treatment for those without. A local survey confirmed that most patients were treated similarly and followed-up routinely clinically and radiographically.

A 5 year review of all notes was performed and a clinical or telephone review achieved in 80%. No affected hip developed a slipped upper femoral epiphysis or Perthes' disease although in one child the contralateral hip developed Perthes' disease. Recurrent admissions with TS occurred in 12%. In 3 patients, the diagnosis of TS was changed to juvenile chronic arthritis and a further 3 cases re-presented with other pathology which accounted for their pain.

TS remains a diagnosis made by exclusion of other pathology but it is a discrete entity. Basic blood tests and an ultrasound scan will help identify patients who can be treated at home. Routine clinical and radiological follow-up is not required.

### THE LONG TERM RESULTS OF THE DILLWYN EVANS COLLATERAL PROCEDURE FOR RELAPSED CLUB FOOT

***C. M. Dent, G. P. Graham**

The purpose of the study was to determine the long term outcome of the Dillwyn Evans procedure for relapsed club feet and to establish whether the results deteriorate with time. Sixty feet in 45 patients average age 28.8 years were reveiwed at an average 22.6 years post-operatively. The feet were scored using five scoring systems, looking at function and deformity. Seven (12%) of the 60 feet had required triple fusion at skeletal maturity. Four feet (7%) required a repeat Dillwyn Evans procedure at an early stage. Six feet (11%) had deteriorated in the last ten years. However in three of these this was attributable to pre-existing joint damage rather than to time alone. Good or satisfactory results were scored by 60-80% of feet depending on the scoring system used. 90% of the patients were able to pursue unrestricted activities. In conclusion the rate of degenerative change and functional deterioration with time is low unless there is pre-existing joint damage. A scoring system based on function rather than deformity is more appropriate in this older age group.

### GOLFERS ELBOW

***Kevin J. O'Dwyer, Colin R. Howie**
Princess Elizabeth Orthopaedic Hospital, Exeter

A retrospective study of 95 cases of medial epicondylitis in 83 patients is reported. This represented 9 percent of patients referred with epicondylitis over a 20 year period. Most cases were work related (90 percent) whilst sporting or leisure activities played a significant role in only 10 percent. 7 percent had recovered between referral and the time of surgical consultation. Whilst the majority of patients settled on conservative treatment, surgical intervention was necessary in resistant cases and accounted for 12 percent of our series. This compared with an operative rate of under 4 percent in patients with lateral epicondylitis in the same period. When necessary, common flexor origin release should be performed without exploration of the ulnar nerve, as this may lead to ulnar neuritis secondary to perineural scarring. The results of surgical treatment for resistant cases of this condition were good with only one poor long term result though keloid scarring and a dimple related to the release were noted.

### POSTERIOR SPINAL FUSION WITH SCREW FIXATION

***V. Seal**
Hereford

Chronic low back pain is a common and difficult condition to treat. Patient selection and operative technique remains debatable.

**Patient selection** — daily disabling lumbosacral pain with discographically proven degenerative disc disease and no previous spinal surgery.

**Operative technique** — interlaminar fusion. Screw fixation of facet joints. No distant bone graft. Early mobilisation. No external brace support. First review undertaken at 7 years indicating an 84% successful outcome for single level fusion and 80% for double level. Second review undertaken at 11 years indicating an 84% successful outcome for single level and 77% otucome for double level.

**Clinical Material** — 101 patients (60 female) followed for 12 years — 1978-1990. The back pain occurred spontaneously in 49%, a domestic injury in 21%, an injury in leisure in 13% and industrial injury 17%. 44% had permanent pain from the outset and 56% intermittent pain becoming permanent. 20% of patients had normal plain radiographs. 50 patients — 1 level fusion and 51, 2 level. 62 patients replied to a detailed questionnaire, 35 assessments were made from the notes and 4 patients were lost to follow up.

**Results**

**Single level** — pain significantly improved — 72%.
Improved physical activity — 66%
**Double level** — pain level reduced — 82%
Physical activity increased — 71%.
71% returned to work.
A compensation claim was involved in 6 patients.

**Failure** — 21, equal percentage distribution male and female
Single level 28% Double level 16%
**Success at pseudarthrosis repair** — 13 patients had a positive psychiatric history.
In 9 (69%) the result was considered a success.
**Subjective assessment** — Single level — very pleased 36%
pleased 36%
Indifferent or displeased 28%
**Subjective assessment** — Double level — very pleased 35%
pleased 49%
Indifferent or displeased 16%

The analysis of the results of 19 private patients indicates success in 79%.

Posterior spinal fusion with screw fixation and early mobilisation without external support can be associated with significant lessening of pain in 80% of patients and improvement of physical activities rather less. Industrial accidents were not common in this series. The results in private patients, NHS patients and patients with proven previous psychiatric history were similar.

### FIXED MINIDOSE WARFARIN PROPHYLAXIS IN TOTAL HIP REPLACEMENT

**(Paper presented at a previous meeting in Cheltenham)**

***M. J. F. Fordyce, A. S. Baker, G. Stadden**
Winford Orthopaedic Hospital, Bristol

The incidence of thromboembolism is particularly high following total hip replacement (THR). Deep vein thrombosis (DVT) occurs in 40-70% of patients and pulmonary embolism detected by scintigraphy in 20-25%. It is not known how many patients eventually suffer the post-phlebitic limb syndrome but 1-2% die from pulmonary embolism.

Many prophylactic regimens have been described but none have proved ideal.

A recent report has shown a significant reduction in DVT formation in patients undergoing gynaecological operations, using a fixed peri-operative low dose of warfarin. This regimen has the advantages that there were no bleeding or wound healing complications and the patients could continue their prophylaxis after their discharge and return home.

The aim of this study was to perform a prospective, double-blind, placebo controlled trial to assess the thrombo-prophylactic efficacy of this simple regimen in patients undergoing total hip replacement.

One hundred and forty-eight patients were randomly allocated to receive either one milligram of warfarin daily for one week prior and three weeks after surgery or a placebo for the same period.

The patients were well matched for age, sex, obesity score, type of arthritis and pre-operative activity.

All patients had their operation performed in the lateral decubitus position under a standardised general anaesthetic.

DVT was diagnosed using the 1-125 fibrinogen uptake method.

Blood tests were performed to establish any alteration in clotting function during the trial period. Blood loss was measured. The wounds were examined clinically and by ultrasound for wound healing problems or haematoma formation.

All patients were reviewed six weeks post-operatively to establish whether any thrombo-embolic complications had occurred since discharge.

Results show:

1. There was no difference in clotting function, blood loss, wound healing or haematoma formation.
2. No significant difference in DVT formation or the occurrence of pulmonary embolism between the two groups.

In conclusion, we have shown that fixed minidose warfarin has no thromboprophylactic effect in patients undergoing total hip replacement and its use cannot be recommended.

### THE CLINICAL BIOMECHANICAL ASPECTS OF GORETEX PROSTHETIC CRUCIATE LIGAMENT REPLACEMENT 6.5 YEAR EXPERIENCE (Poster)

***V. Seal**
Hereford

**Material**
62 Goretex expanded PTFE cruciate ligament prostheses have been studied prospectively from 2.5 to 6.5 years. Follow up — 88%.
**Biology**
Effusions (13-21%). 12 short lasting. 1 persistent. 7 post traumatic. 5 spontaneous. 4 recurrent.
Aspirate — histiocytic response.
Synovium — patchy mild synovitis. PTFE particles with polarised light microscopy. No chronic synovitis.
**Bone Ingrowth**
In bone tunnels, minimal and peripheral bone ingrowth. Dense fibrosis at entrance and exit of bone tunnels universal.
**Mechanical**
Fixation. No failure at eyelets or screws.
Failure. 11 (18%) proven prostheses ruptured. 6 with further injury. 5 spontaneous. Clinically assumed but not proven failure — 12 (19.5%). 5 asymptomatic. Proven slack but intact prostheses (symptomatic) — 2. Thus assumed total failure — 25 (40%). Revision — 14 (23%). Failure occurred at the deep exit of the femoral tunnel and within the intercondylar notch but not within the bone tunnels. Laxity signs increase a little with time.
**Return to Sport**
70% of those wishing to do so returned to sport for 13 months to 6.5 years post operatively. 10 ruptured prostheses with further sports injury.
**Compact Diameter Prosthesis**
67 patients 0-2.5 years, 100% review.
**Biology**
As above — Effusions. 6 (9%). 4 resolving. 2 persistent.
**Mechanical**
Proven failure — 0. Seemed failure — 2 (3%). Total assumed failure — 2 (3%). Revision — 0.
**Return to Sport**
78% playing sport up to 2.5 years post operatively.
**European/Canadian Prospective Trial with Compact Diameter Ligament in Third Prospective Year**
Participants meet annually to discuss prospective results.

### A COMPLETE INTRA-ARTICULAR FAT PAD AROUND THE HUMAN PATELLA (Poster)

***R. L. M. Newell**
Department of Anatomy, University of Wales College of Cardiff

The infrapatellar pad of the human knee is well described in anatomical texts, and the presence of a suprapatellar pad is used by radiologists in diagnosing small effusions. This demonstration is based on a study of 21 knees from unselected dissecting room cadavers. It points out that the infrapatellar and suprapatellar pads are linked along the medial and lateral borders of the patella to form a continuous circumferential fat pad. The name *corona adiposa patellae* is proposed for this structure. The pad forms a flexible part of the patellofemoral articulation, accommodating to movements of the joint and occupying the space around the articular margin of the patella. It sometimes overlaps the patellar articular cartilage and forms a pouch in which small loose bodies may lodge and evade discovery at arthroscopy.

